# The Antifungal Activity and Mechanism of Dehydroabietic Acid Against *Alternaria alternata* Causing Poplar Leaf Spot

**DOI:** 10.3390/jof11040265

**Published:** 2025-03-28

**Authors:** Yun-Ze Chen, Yun-Di Zhang, Cheng Chen, Qiu-Er Sa, Jing Yang, Guo-Cai Zhang

**Affiliations:** 1 School of Biological Sciences, Guizhou Education University, Wudang District, Guiyang 550018, China; chenyunze@gznc.edu.cn; 2 Heilongjiang Province Key Laboratory of Forest Protection, School of Forest, Northeast Forestry University, Hexing Road 26, Xiangfang District, Harbin 150040, China; 13251611896@163.com (Y.-D.Z.); chengc58@126.com (C.C.); sa648036466@126.com (Q.-E.S.); zhang640308@126.com (G.-C.Z.); 3 College of Forestry, Guizhou University, Huaxi District, Guiyang 550025, China

**Keywords:** dehydroabietic acid, *Alternaria alternata*, antifungal mechanism, leaf spot of poplar

## Abstract

Dehydroabietic acid (DHA) is a secondary metabolite isolated from rosin, which has certain antifungal activity, but its inhibitory effects against *Alternaria alternata* are unclear. In the present study, we found that DHA inhibited the mycelial growth of *A. alternata*, *Botrytis cinerea*, *Valsa mali*, *Pestalotiopsis neglecta*, and *Fusarium oxysporum* in a concentration-dependent manner, with the best inhibitory effect against *A. alternata*. Moreover, DHA can also inhibit the spore germination of *A. alternata*. Then, in vivo inoculation experiments showed that the leaf lesions of *Populus alba* gradually decreased with the increase in DHA concentration. The disease of *P. alba* leaves inoculated with *A. alternata* was not obvious after treatment with 800 mg/L DHA. The scanning electron microscopy showed that the mycelial morphology was abnormal, with crinkles and depressions. Meanwhile, the relative conductivity, soluble protein content, malondialdehyde content and hydrogen peroxide content of *A. alternata* were significantly increased after DHA treatment, which affected the integrity of the cell membrane and increased the permeability of *A. alternata*, resulting in a large leakage of intracellular substances, exacerbating the degree of lipid peroxidation of the cell membrane of *A. alternata* and causing oxidative damage to cells. The enzyme activity assay showed that treatment with 56.015 mg/L (EC_50_) DHA significantly reduced the activities of antioxidant enzymes (superoxide dismutase, catalase, peroxidase) and cell-wall-degrading enzymes (endoglucanase, polygalacturonase, pectin lyase) in *A. alternata* (*p* < 0.05), resulting in a decrease in the activity of pathogenic fungi, as well as a reduction in the ability of the *A. alternata* to degrade the cell wall of the host plant, which led to a decrease in the ability of the *A. alternata* to infest the host plant. Moreover, the decrease in the relative expression of defense-related enzyme genes (*AaSOD, AaPOD, AaCAT*) and pathogenicity-related enzyme genes (*AaPL, AaPG*) was consistent with the enzyme activity results. Thus, the present study revealed the fungicidal activity and mechanism of DHA against *A. alternata* and the potential of DHA to be developed as a plant-derived antifungal agent was established.

## 1. Introduction

*Populus* spp. (poplar), a keystone genus in global afforestation programs, plays a vital role in ecological restoration, soil conservation, and sustainable timber production due to its rapid growth and environmental adaptability [1,2]. However, poplar cultivation faces persistent phytopathological threats, with over 300 documented fungal pathogens causing diseases ranging from stem cankers to foliar necrosis [3]. Among these, leaf spot disease has emerged as a particularly destructive issue in northeastern China, severely affecting nursery stocks of economically important species including *P. alba*, *P. tomentosa*, *P. simonii*, and *P. pseudosimonii* [4]. Field observations reveal characteristic symptoms of brown necrotic lesions on foliar tissues, which progress to premature defoliation, growth retardation, and irreversible wood quality degradation under epidemic conditions. The pathogenicity of this foliar disease has been conclusively linked to *A. alternata*, a cosmopolitan asexual fungus renowned for its broad host range spanning 380+ plant species across agricultural and forestry systems [5,6]. As a polyphagous pathogen, *A. alternata* exhibits exceptional ecological plasticity, capable of infecting diverse plant organs including roots, stems, leaves, and fruits, where it induces tissue maceration, blight, and rot [7,8]. Its infection mechanisms in poplar involve the production of host-specific toxins and cell-wall-degrading enzymes, enabling rapid colonization of leaf parenchyma and vascular tissues [4]. Current management strategies predominantly rely on synthetic fungicides such as mancozeb, daconil, and captafol. While long-term use of fungicides causes pesticide residues as well as resistance problems, which have a negative impact on human health and the environment [9,10,11]. Given the various drawbacks of using chemical pesticides, as well as the strengthening of people’s awareness of environmental protection, the trend of modern pesticide development tends to develop new highly efficient, low-toxicity and environmentally friendly pesticides, and people are searching for new substitutes in renewable natural resources.

Rosin is a renewable natural advantageous resource obtained through the distillation process of turpentine [12,13]. Dehydroabietic acid (DHA), also known as dehydrorosin acid, is one of the constituents of rosin and the main component of disproportionated rosin. According to the literature, DHA is an important natural tricyclic diterpene resin acid, which is stable, has strong antioxidant capacity, good biocompatibility and biodegradability [14]. It has a variety of biological activities, such as anti-microbial, insecticidal, anti-carcinogenic, and anti-aging [15,16,17,18]. Soderberg et al. [19] found that DHA has bacteriostatic activity against Gram-positive and Gram-negative bacteria. The inhibitory effect on bacteria may be due to the biofilm-targeted nature of DHA, which inhibits bacterial proliferation and therefore also acts as a natural bacterial biofilm inhibitor [20,21]. Due to the advantages of the structure of DHA, the modification based on the benzene ring and carboxyl group is the current hotspot in the study of DHA [22]. Gao et al. [23] synthesized two series of dehydroabietyl oxime ester derivatives using DHA as the lead compound; an in vitro antifungal test showed that these compounds had a better inhibitory effect on six plant pathogenic fungi—one of the compounds had the strongest activity on tomato gray mold fungus—and further study found that the compound could change the morphology and ultrastructure of *Botrytis cinerea* mycelium, leading to the dysfunction of nucleus and mitochondria, thus causing apoptosis of *B. cinerea* cells, and it had good protective and therapeutic effects on tomato. However, relatively few studies have explored the inhibitory effect of DHA on pathogenic fungi and its mechanism.

Therefore, the present study confirmed that DHA has different inhibitory effects on five common pathogenic fungi and took *A. alternata* as the research object to investigate the inhibitory mechanism of DHA on *A. alternata*, which made up the gap of DHA in this field. Meanwhile, the control effect of DHA on *P. alba* leaf spot provided a theoretical basis for developing DHA as a substitute for chemical pesticides.

## 2. Materials and Methods

### 2.1. Pathogens and Spore Suspensions

The phytopathogenic fungi *A. alternata*, *Pestalotiopsis neglecta*, *B. cinerea*, *Fusarium oxysporum*, and *Valsa mali* were provided by the Key Laboratory of Forest Protection of Heilongjiang Province (Northeast Forestry University, Harbin, China). These pathogenic fungi were cultured on potato dextrose agar (PDA) medium at 25 °C.

The spores of *A. alternata* were collected with reference to the method described by Li et al. [24]. *A. alternata* was inoculated onto PDA medium, and the medium was inverted and incubated in a constant temperature incubator at 25 °C for 7 d under dark conditions. Sterile water was poured into the Petri dishes after 7 d in small amounts and several times, and mycelium and spores were collected by gently scraping the surface of the medium with a sterile inoculation loop, and the mycelium was filtered off using sterilized skimmed cotton to obtain the spore suspension. The concentration of the spore suspension was adjusted to 1 × 10^6^ spores/mL using a haemocytometer plate under a 400× inverted optical microscope.

### 2.2. Reagents and Plant Samples

DHA was purchased from Jingmen Dongxin Biotechnology Co., Ltd., (Jingmen, China). The kits for the determination of superoxide dismutase (SOD), peroxidase (POD), catalase (CAT), endo-β-1,4 glucanase (EG), polygalacturonase (PG), pectin lyase (PL), malondialdehyde (MDA), and hydrogen peroxide (H_2_O_2_) were purchased from Suzhou Grace Biotechnology Co., Ltd. (Suzhou, China). The RNA extraction kit was RNA pure Plant Kit (DNase I), purchased from Jiangsu Kangwei Century Biotechnology Co., Ltd. (Taizhou, China). The reverse transcription reagent was Prime Script^TM^ RT reagent Kit with gRNA Eraser (Perfect Real Time), and the fluorescence quantification kit was TB Green Premix Ex Taq^TM^ II (Tli RNase H Plus), both of which were purchased from Beijing TaKaRa Biotechnology Co., Ltd. (Beijing, China). Other analytical grade reagents and solvents were obtained from Tianjin Fuyu Fine Chemical Co., Ltd. (Tianjin, China) and were not further purified before use.

The poplar leaves used in the experiment were collected in the on-campus forest of Northeast Forestry University, from which healthy, intact and similar-sized leaves were selected.

### 2.3. Effect of DHA on Mycelial Growth of Five Pathogenic Fungi

The inhibitory effect of DHA on five pathogenic fungi was determined with reference to the mycelial growth inhibition method described by Li et al. [25]. DHA was added to the PDA medium using DMSO as a co-solvent (final concentration of 2%) such that the final concentration of DHA was 800, 400, 200, 100, 50, 25, and 12.5 mg/L. The medium with equal amounts of sterile water and DMSO was used as a blank and solvent control. Mycelial plugs 5 mm in diameter were inoculated in the center of 90 mm diameter Petri dishes, then sealed and inverted for incubation in a constant temperature incubator at 25 °C. The radial growth diameter of the pathogen was determined when the control group was fully grown and the mycelial growth inhibition rate was calculated, which was repeated three times for each group. The growth inhibition rate (%) was calculated as follows:

$$Inhibition\;rate\;\left(\%\right)=\frac{Growth\;diameter\;in\;control-Growth\;diameter\;in\;treatment}{Growth\;diameter\;in\;control-0.5}\times 100$$

### 2.4. Effect of DHA on Spore Germination of A. alternata

The effect of DHA on spore germination of *A. alternata* was investigated using the suspension-drop method described by Qiu et al. [26]. A 20 μL suspension of spores was mixed with 20 μL of a solution containing DHA at concentrations of 9.966 mg/L (EC_30_) and 56.015 mg/L (EC_50_), and the control group was added with equal amounts of sterile water and DMSO. After dropping 40 μL of liquid into the depression of the haemocytometer plate and incubating in the constant temperature incubator at 25 °C for 24 h, the number of spore germination was observed randomly in five fields of view using a microscope, and the inhibition of spore germination was calculated, which was repeated three times for each group. Spore germination rate (%) and germination inhibition rate (%) were calculated as follows:

$$Spore\;germination\;rate\;\left(\%\right)=\frac{Number\;of\;germinated\;spores}{Total\;number\;of\;observed\;spores}\times 100$$

$$Spore\;germination\;inhibition\;rate\;\left(\%\right)=\frac{Germination\;rate\;in\;control-Germination\;rate\;in\;treatment}{Germination\;rate\;in\;control}\times 100$$

### 2.5. In Vivo Control Effect

The leaves were surface-disinfected in a 1% sodium hypochlorite solution for 45 s, rinsed in sterile water, soaked in a 75% ethanol solution for 30 s, and rinsed twice in sterile water. After being cleaned and sterilized, the surface of each leaf was pricked three times using a sterilized inoculation needle, and then each leaf was sprayed with 5 mL of DHA solution (containing 2% DMSO), with a DHA concentration of 56.015 mg/L (EC_50_), 400 mg/L, and 800 mg/L, and the control group was sprayed with 2% DMSO solution. After the air-drying of the medicinal solution on the surface of *P. alba* leaves, *A. alternata* mycelial plugs (7.5 mm in diameter) were inoculated at the punctures of the leaves and wrapped around the petioles using sterilized skimmed cotton dipped in sterile water to provide moisture, and the inoculated leaves were moisturized and cultured in a thermostatic incubator at 25 °C [27]. The onset of disease was observed after 5 d of incubation and the lesion area was determined using Image J 1.54k. Three replicates were set up for each group of treatments, with five leaves in each replicate.

### 2.6. Morphological Observation of A. alternata Mycelium

Scanning electron microscopy (SEM) was used to study the effect of DHA on mycelial morphology during the growth of *A. alternata*. Mycelial plugs (7.5 mm in diameter) were inoculated on PDA plates containing EC_50_-level DHA, with parallel controls using untreated PDA for blank and DMSO-contained PDA for solvent effects. Sterilized coverslips were inserted obliquely into the medium at an angle of 45°, about 2–3 cm from the centrally positioned mycelial plugs, sealed and incubated in an inverted position in a constant temperature incubator at 25 °C, modifying the method described by Li et al. [28]. When the mycelium grew onto the coverslips, the coverslips with attached hyphae were removed and immediately immersed in 2.5% (*v*/*v*) glutaraldehyde PBS fixative for 4 h at room temperature. After fixation, the specimens were rinsed with PBS buffer of identical concentration, followed by sequential dehydration through an ethanol gradient series (30%, 50%, 70%, 80%, 90%, 95%, and 100%) prepared in PBS. Subsequently, the samples underwent vacuum freeze-drying for 12 h before being mounted, sputter-coated with gold-palladium, and examined under a SEM at 5000× magnification.

### 2.7. Effect of DHA on Extracellular Proteins and Electrical Conductivity in A. alternata

A mixture of DHA dissolved in DMSO was added to the PDB medium (total system 100 mL) with final concentrations of DHA of 9.966 mg/L (EC_30_) and 56.015 mg/L (EC_50_). Erlenmeyer flasks containing PDB medium were filled with an equal amount of sterile water as a blank control and an equal amount of DMSO as a solvent control. Then, five mycelial plugs of *A. alternata* (5 mm in diameter) were placed in Erlenmeyer flasks, sealed and placed in an oscillating incubator at 25 °C and 150 rpm, and three replicates were set up for each group of treatments, and the mycelial samples and supernatants were collected at 48 h, 54 h, 60 h, 66 h, and 72 h after incubation, respectively. Mycelium samples were filtered using sterile silk cloth, rinsed three times with 0.9% NaCl solution, and then stored at −80 °C after blotting with sterile filter paper. The supernatant obtained after centrifugation of the filtrate was used for subsequent experimental determinations and was also stored at −80 °C.

The supernatant was removed to measure the extracellular conductivity (μS/cm) of *A. alternata* using the DDS-11 digital conductivity meter (Shanghai Precision Scientific Instrument Co., Ltd., Shanghai, China) and soluble protein leakage was determined according to the method described by Bradford [29]. Three replicates were set for each group of treatments.

### 2.8. Effect of DHA on MDA and H_2_O_2_ in A. alternata

To assess the extent of *A. alternata* cell membrane damage by DHA, the content of malondialdehyde (MDA) and hydrogen peroxide (H_2_O_2_) was determined in *A. alternata* using the corresponding kits, respectively. Samples of *A. alternata* mycelium were prepared according to the method mentioned in Section 2.7, processed according to the instructions of the kit, and then set at the corresponding wavelengths using a Super Max 3000 AL multifunctional microplate reader (Shanghai Shanpu Biotechnology Co., Ltd., Shanghai, China) to read the absorbance values, and then calculate the contents of MDA and H_2_O_2_. Three replicates were set for each group of treatments.

### 2.9. Effect of DHA on the Enzyme Activities

The mycelium samples and supernatant samples of *A. alternata* were prepared according to the Section 2.7 and operated according to the instructions of the enzyme activity determination commercial kits of SOD, POD, CAT, EG, PG and PL. SOD, POD and CAT activities were determined using *A. alternata* mycelium samples, and EG, PG, and PL activities were determined using *A. alternata* supernatant samples. The Super Max 3000 AL multifunctional microplate reader (Shanghai Shanpu Biotechnology Co., Ltd.) was used for the determination of antioxidant enzymes and cell-wall-degrading enzymes activities in *A. alternata*, and three replicates were set up for each group of treatments.

### 2.10. Effect of DHA on the Gene Expression

Samples of *A. alternata* mycelium cultured for 72 h were prepared according to 2.6, total RNA of *A. alternata* was extracted using the corresponding kit, and reverse transcription was performed to synthesize cDNA, which was stored at −20 °C for subsequent experiments. The gene sequences were obtained from the NCBI website, *AaBenA* was selected as the reference gene, and primer design was performed using Bioxm 2.7.1 (Table 1). Changes in the expression of defense enzyme genes (*AaSOD*, *AaPOD*, *AaCAT*) and pathogenic enzyme genes (*AaPL*, *AaPG*) in *A. alternata* were determined using the CFX96 Real-Time PCR System (Bio-Rad Laboratories, Hercules, CA, USA) according to the instructions of the quantification kit, and three independent biological replicates were performed for each treatment. The expression of the tested genes was calculated using the 2^−ΔΔCT^ method [30].

### 2.11. Statistical Analysis

The significance of differences was analyzed by one-way ANOVA using SPSS 26.0 software (SPSS Inc., Chicago, IL, USA) and *p* < 0.05 was considered statistically significant, while the toxicity equations as well as EC_30_ and EC_50_ concentrations were calculated by probit analysis. GraphPad Prism 8.0 (GraphPad Software Inc., San Diego, CA, USA) was used for graphics creation.

## 3. Results

### 3.1. Effect of DHA on Mycelial Growth Inhibition of Five Pathogenic Fungi

**Figure 1:**
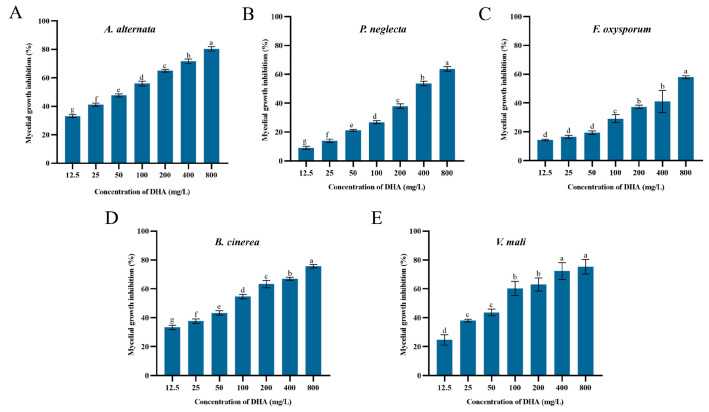
The inhibition rate of the mycelial growth of *A. alternata* (**A**), *P. neglecta* (**B**), *F. oxysporum* (**C**), *B. cinerea* (**D**), *V. mali* (**E**) by different concentrations of dehydroabietic acid. The bars represent the standard error of the mean (*n* = 3), and the letters a–g indicate significant differences between different concentrations (*p* < 0.05).

The inhibitory effects of DHA on the mycelial growth of five pathogenic fungi are shown in Figure 1. DHA showed concentration-dependent inhibitory effects on different forest pathogenic fungi, and there was a certain degree of variability between different concentrations (*p* < 0.05). Among them, DHA showed the best inhibitory effect on *A. alternata* and the worst inhibitory effect on *F. oxysporum*. When the concentration of DHA was 800 mg/L, the inhibition rates of *A. alternata*, *B. cinerea*, *V. mali*, *P. neglecta* and *F. oxysporum* were 80.33%, 75.67%, 75.33%, 63.76% and 57.94%, respectively.

Probit analysis was performed using SPSS 21.0 to determine the toxicity equations of DHA against the five pathogenic fungi as well as the values of EC_50_ and EC_30_ (Table 2). The toxicity equation of DHA against *A. alternata* was Y = 0.7X − 1.23 (R^2^ = 0.991), EC_30_ against *A. alternata* mycelial growth was 9.966 mg/L, and the value of EC_50_ was 56.015 mg/L, which were lower than those of the other four pathogenic fungi. Therefore, DHA had an inhibitory effect on all five pathogenic fungi, but the best inhibitory effect on *A. alternata*.

### 3.2. Effect of DHA on Spore Germination of A. alternata

**Figure 2:**
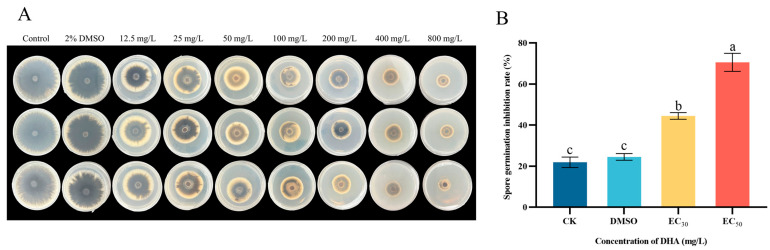
The inhibitory effect of different concentrations of dehydroabietic acid on mycelial growth (**A**) and spore germination (**B**) of *A. alternata*. (**A**) The inhibition of mycelial growth of *A. alternata* after different concentrations of DHA; (**B**) The inhibition of *A. alternata* after treatment with EC_30_ and EC_50_ concentrations of DHA. The bars represent the standard error of the mean (*n* = 3), and the letters a–c indicate significant differences between different concentrations (*p* < 0.05).

The effects of DHA on the mycelial growth and spore germination of *A. alternata* at different concentrations are shown in Figure 2. Compared with the control group, the growth of *A. alternata* mycelium in the DHA-treated group was significantly inhibited (*p* < 0.05), and the mycelium in the treated group grew slowly, the color changed, and the inhibitory effect gradually increased with the increase in DHA concentration (Figure 2A). DHA could significantly inhibit the germination of *A. alternata* spores (*p* < 0.05), and the inhibition effect was concentration-dependent, and the inhibition rate of DHA on the spore germination of *A. alternata* was 44.49% and 70.55% at the concentrations of EC_30_ and EC_50_ (Figure 2B).

### 3.3. In Vivo Control Effect

**Figure 3:**
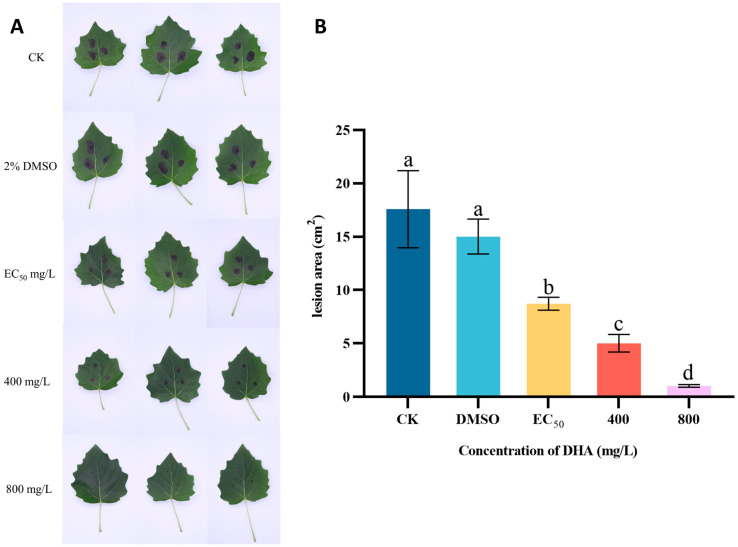
In vivo effect of different concentrations of dehydroabietic acid on poplar leaf spot. (**A**) The disease symptoms of *P. alba* leaves inoculated with *A. alternata* after spraying different concentrations of DHA. (**B**) The lesion areas of *P. alba* leaves inoculated with *A. alternata*. The bars represent the standard error of the mean (*n* = 3), and the letters a–d indicate significant differences between different concentrations (*p* < 0.05). CK, a blank control treated with sterile water.

The protective effect of DHA on *P. alba* leaves was investigated through the triple interaction of DHA-*P. alba* leaves-*A. alternata* (Figure 3). The disease severity of *P. alba* leaves inoculated with *A. alternata* after DHA treatment is shown in Figure 3A. In the control group, the poplar leaves inoculated with *A. alternata* showed irregular brown spots with a large area and high severity, and when 800 mg/L DHA was used for the treatment, the inoculation position lesions of poplar leaves were not obvious and showed almost no disease. After measuring the lesion area, it was found that there was a significant difference between the disease area of *A. alternata* inoculated *P. alba* leaves treated with different concentrations of DHA and that of the control group (*p* < 0.05), and the lesion area gradually decreased with the increase in the concentration of DHA; the lesion area of the blank control group was about 17.59 cm^2^. When the concentration of DHA was EC_50_, the disease area was 8.72 cm^2^, which decreased by 50.43% compared with the control group, and when the concentration of DHA was 800 mg/L, the disease area was 1.03 cm^2^, which decreased by 94.14% compared with the control group (Figure 3B). It can be seen that DHA can inhibit the infestation of *A. alternata* on *P. alba* leaves and reduce the incidence area of poplar leaves, which has a desirable protective effect.

### 3.4. Effect of DHA on Mycelial Morphology

**Figure 4:**
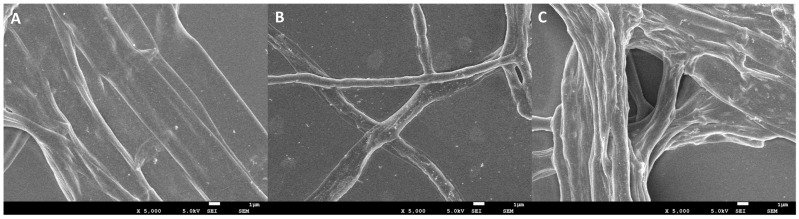
SEM images. (**A**) Mycelial morphology of *A. alternata* cultured under normal conditions (blank control); (**B**) mycelial morphology of *A. alternata* after treatment with DMSO (solvent control); (**C**) mycelial morphology of *A. alternata* after treatment with 56.015 mg/L (EC_50_) DHA. Bar = 1 μm.

To further observe the effect of DHA on the morphology and structure of *A. alternata* mycelia, the morphology of mycelia in the blank control group, solvent control group, and the DHA-treated group (EC_50_) was observed by SEM (Figure 4). The results showed that both the mycelium of the two control groups were thick and full, with a smooth surface and normal morphology and structure (Figure 4A,B), but after DHA treatment, the mycelium structure was abnormal, and the surface of the mycelium became rough and twisted and appeared to be wrinkled and concave (Figure 4C). Therefore, it can be shown that DHA can destroy the normal morphology and structure of mycelium, thus affecting the normal growth of *A. alternata*.

### 3.5. Effect of DHA on Extracellular Proteins and Conductivity of A. alternata

**Figure 5:**
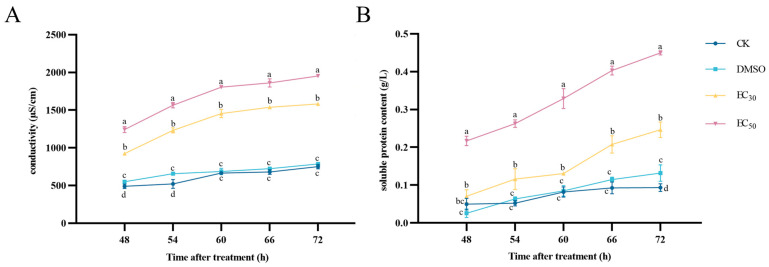
The effect of dehydroabietic acid at different concentrations (0 mg/mL and DMSO as control, EC_30_, and EC_50_) on the extracellular conductivity (**A**) and soluble protein content (**B**) of *A. alternata*. The bars represent the standard error of the mean (*n* = 3), and the letters a–d indicate significant differences between different concentrations (*p* < 0.05).

Conductivity measurements can be used to assess the changes in the permeability of the cell membrane of pathogenic fungi, the changes in the extracellular conductivity of *A. alternata* under different concentrations of DHA treatment are shown in Figure 5A. With the increase in the incubation time, the conductivities of the experimental groups showed an increasing tendency, but that of the control group was slowly increased and maintained at a lower level. The conductivity of the EC_50_-treated group reached a maximum of 1953.67 μS/cm at 72 h of incubation, which was 1.24 times higher than that of the EC_30_-treated group, and 2.6 times higher than that of the blank control group. Therefore, DHA caused damage to the cell membrane structure of *A. alternata*, leading to an increase in cell membrane permeability and extravasation of intracellular substances, which resulted in an increase in conductivity.

With the increase in treatment time, the extracellular protein content of *A. alternata* showed a rising trend in all experimental groups, the extracellular protein content of mycelium treated with DHA at concentrations of EC_30_ and EC_50_ was higher than that of the blank control group and the DMSO control group, and the higher the concentration was, the higher the extracellular protein content was (Figure 5B). At 72 h of incubation, the protein content of EC_30_ and EC_50_ treatment groups were 2.62 and 4.78 times higher than that of the blank control group, so it can be seen that DHA caused the abnormal cell membrane of *A. alternata* in a concentration-dependent manner, and the leakage of soluble protein occurred.

### 3.6. Effect of DHA on MDA and H_2_O_2_ Contents of A. alternata

**Figure 6:**
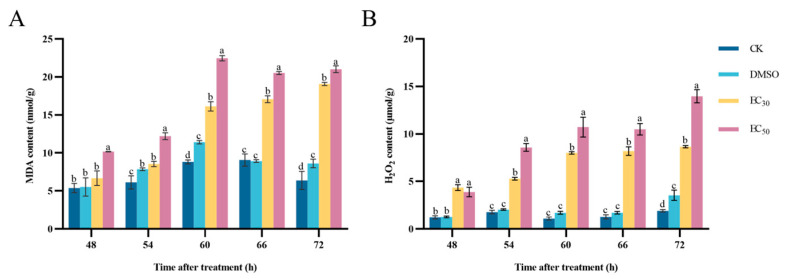
The effect of dehydroabietic acid at different concentrations (0 mg/mL and DMSO as control, EC_30_, and EC_50_) on the MDA content (**A**) and H_2_O_2_ content (**B**) of *A. alternata*. The bars represent the standard error of the mean (*n* = 3), and the letters a–d indicate significant differences between different concentrations (*p* < 0.05).

The peroxidation of cell membranes occurs, resulting in the production of MDA. The MDA content in the pathogens treated with EC_30_ and EC_50_ concentrations of DHA was higher than that in the control group. At 48–60 h of incubation, the MDA content in the treated groups showed an increasing trend, after which it gradually stabilized. The highest content of the EC_50_-treated group was 22.46 nmol/g at 60 h of incubation, which was 1.39 times higher than that of the EC_30_-treated group and 2.55 times higher than that of the blank control group (Figure 6A). Therefore, the treatment of DHA led to a large accumulation of MDA in *A. alternata*, and this result also indicates a deepening of lipid peroxidation of the cell membrane of the *A. alternata* and a further increase in the oxidative damage of the cell membrane.

H_2_O_2_ is one of the most abundant reactive oxygen species (ROS) in the cell, which can be used as a key signaling molecule to regulate the growth and development of organisms and resist adversity stress. The H_2_O_2_ content in *A. alternata* after DHA treatment increased with the increase in incubation time, and the H_2_O_2_ content in mycelium reached the maximum at 72 h of incubation, and the H_2_O_2_ content in the EC_50_-treated group was 1.61 times higher than that in the EC_30_-treated group and 7.29 times higher than that in the blank control group (Figure 6B). It can be seen that the DHA can lead to the increase in H_2_O_2_ content in *A. alternata*, and when H_2_O_2_ accumulates to a certain extent it will cause some oxidative damage to the cells.

### 3.7. Effect of DHA on Antioxidant Enzyme Activities of A. alternata

**Figure 7:**
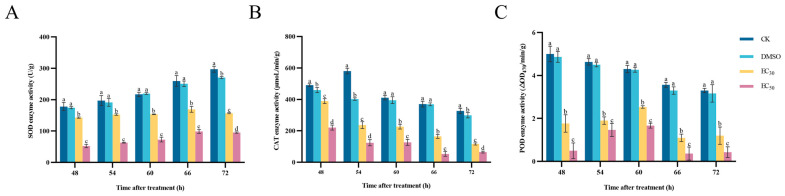
The effect of dehydroabietic acid at different concentrations (0 mg/mL and DMSO as control, EC_30_, and EC_50_) on the SOD activity (**A**), CAT activity (**B**) and POD activity (**C**) of *A. alternata*. The bars represent the standard error of the mean (*n* = 3), and the letters a–d indicate significant differences between different concentrations (*p* < 0.05).

The results of the determination of the effect of DHA on the enzyme activities of SOD, CAT and POD in *A. alternata* are shown in Figure 7. In all experimental groups, the activity of SOD enzyme in *A. alternata* showed an overall increasing trend with the increase in incubation time. The higher the concentration of DHA, the lower the enzyme activity of SOD, and the enzyme activity of the control group was always higher than that of the treated group, and there was a significant difference (*p* < 0.05). After 72 h of incubation, the enzyme activity of SOD in the blank control group was 1.89 times higher than that in the EC_30_-treated group and 3.11 times higher than that in the EC_50_-treated group (Figure 7A).

After treatment with DHA at concentrations of EC_30_ and EC_50_, the CAT enzyme activity in *A. alternata* was always lower than that of the control group and was significantly different from the control group (*p* < 0.05). With the increase in incubation time, the CAT enzyme activity gradually decreased, and when incubated for 66 h, the CAT activity of the EC_50_-treated group was maximally inhibited to 53.28 μmoL/min/g, and the enzyme activity of the blank control group was 370.19 μmoL/min/g, which was 6.95 times higher than that of the EC_50_-treated group. (Figure 7B).

With the increase in incubation time, the POD activity in mycelium under the treatment of EC_30_ and EC_50_ concentrations showed an increase and then a decrease, and gradually stabilized after 66 h. When incubated for 66 h, EC_50_-treated group showed the lowest POD activity, which was 89.4% lower compared with the blank control group (Figure 7C). Therefore, it can be seen that DHA can significantly inhibit the activity of antioxidant enzymes in *A. alternata*, which reduces the pathogen’s own defense ability and thus exerts an inhibitory effect.

### 3.8. Effect of DHA on Cell-Wall-Degrading Enzymes Activities of A. alternata

**Figure 8:**
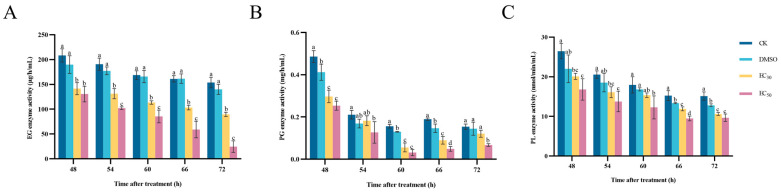
The effect of dehydroabietic acid at different concentrations (0 mg/mL and DMSO as control, EC30, and EC50) on the EG activity (**A**), PG activity (**B**) and PL activity (**C**) of *A. alternata*. The bars represent the standard error of the mean (*n* = 3), and the letters a–d indicate significant differences between different concentrations (*p* < 0.05).

The results of the determination of the effect of DHA on the enzyme activities of EG, PG and PL of *A. alternata* are shown in Figure 8. With the increase in incubation time, the enzyme activity of EG showed an overall decreasing trend, and the EC_50_-treated group had the lowest EG activity. When incubated for 72 h, the activity of EG at EC_50_ concentration was 24.75 μg/h/mL, and that of the blank control group was 153.84 μg/h/mL, which was 6.22 times higher than that of the EC_50_-treated group (Figure 8A).

The higher the concentration of DHA, the stronger the inhibition effect on PG enzyme activity in *A. alternata*, and the PG activity of the blank control group was always significantly higher than that of the EC_50_-treated group (*p* < 0.05). When the incubation time was 60 h, the PG activity of the control group was 4.92 times that of the EC_50_-treated group (Figure 8B).

Compared with the control, DHA was able to significantly reduce the enzyme activity of PL in a concentration-dependent manner. At 66 h of incubation, the lowest PL activity was 9.49 nmol/min/mL in *A. alternata* treated with EC_50_ DHA, and the blank control group was 1.61 times higher than that of the EC_50_-treated group. After that, the PL activity gradually stabilized, but the PL activity of the DHA-treated group was always lower than that of the control group (Figure 8C). It can be seen that DHA can effectively inhibit the activity of cell-wall-degrading enzymes, destroy the infestation ability of *A. alternata*, and decrease the pathogenicity, thus reducing the damage of *A. alternata* to plants.

### 3.9. Effect of DHA on the Gene Expression of Defense-Related Enzymes in A. alternata

**Figure 9:**
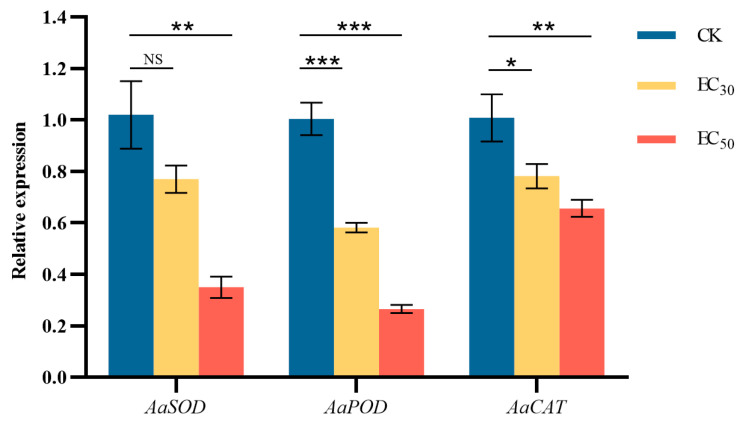
The effect of dehydroabietic acid at different concentrations (0 mg/mL as control, EC_30_, and EC_50_) on the expression of defense-related enzyme genes of *A. alternata*. The bars represent the standard error of the mean (*n* = 3), NS indicates that there is no significant difference in the relative expression of *AaSOD* gene after treatment with EC_30_ concentrations of DHA (*p* > 0.05), the asterisks *, ** and *** indicate significant differences in the relative expression of defense-related enzyme genes after DHA treatment (*p* < 0.05, *p* < 0.01 and *p* < 0.001, respectively).

The effect of DHA on genes encoding *A. alternata* defense-related enzymes was shown in Figure 9. Compared with the control group, genes encoding SOD, POD, and CAT were significantly down-regulated (*p* < 0.05) by DHA treatment, in which the expression of *AaSOD* in the control group was 1.32 and 2.86 times higher than that of the EC_30_ and EC_50_-treated groups, respectively; the expression of *AaPOD* in the control group was 1.73 and 3.76 times higher than that of the treated group, respectively; and the expression of *AaCAT* in the control group was 1.29 and 1.52 times higher than that of the treated group, respectively. The results showed that the expression of the three protective enzyme genes was significantly down-regulated after DHA treatment, but the expression of the gene encoding POD was more obviously regulated, and the lowest expression was found after EC_50_ concentration DHA treatment.

### 3.10. Effect of DHA on the Gene Expression of Pathogenicity-Related Enzyme in A. alternata

**Figure 10:**
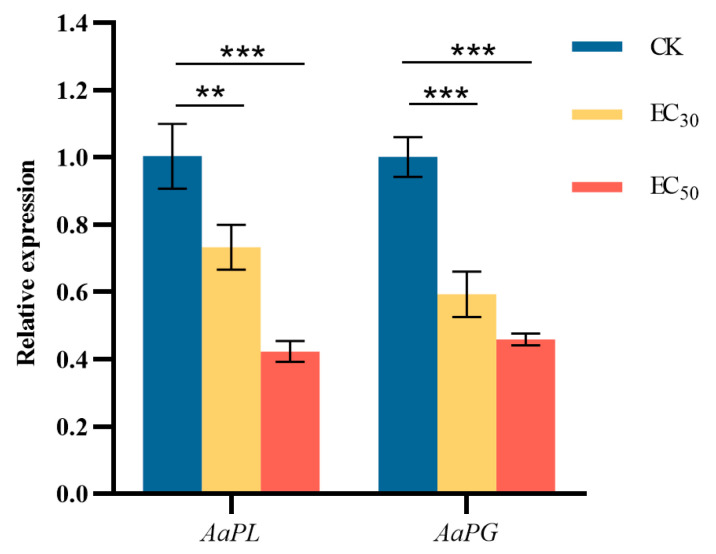
The effect of dehydroabietic acid at different concentrations (0 mg/mL as control, EC_30_, and EC_50_) on the expression of pathogenicity-related enzyme genes of *A. alternata*. The bars represent the standard error of the mean (*n* = 3), the asterisks ** and *** indicate significant differences in the relative expression of pathogenicity-related enzyme genes after DHA treatment (*p* < 0.01 and *p* < 0.001, respectively).

The effect of DHA on genes encoding *A. alternata* pathogenicity-related enzymes was shown in Figure 10. The difference between the expression of genes encoding PL and PG in *A. alternata* after treatment with DHA at the concentration of EC_50_ and that of the control group was highly significant (*p* < 0.001). The expression of *AaPL* in the control group was 1.37 and 2.37 times higher than that in the EC_30_ and EC_50_-treated groups, respectively; and the expression of *AaPG* in the control group was 1.68 and 2.18 times higher than that in the treated groups, respectively. The results showed that DHA treatment could significantly inhibit the expression of the two pathogenic enzyme genes, and the higher the concentration of DHA, the lower the relative expression of *AaPL* and *AaPG*.

## 4. Discussion

Fungal diseases cause huge economic losses to global agriculture and forestry every year. Although the widespread use of chemical pesticides is the main way to control fungal diseases in production, the overuse of chemical pesticides poses a threat to the ecosystem and human health, and the development of plant-derived pesticides will help to reduce the negative impacts of chemical pesticide-induced residues, environmental pollution, and drug resistance [31,32,33]. DHA, a natural product of rosin, can be isolated from resinous acids, which have some antifungal activity in their own right [34]. In the present study, DHA was found to have different levels of fungicidal activity against *A. alternata*, *P. neglecta*, *B. cinerea*, *F. oxysporum*, and *V. mali*. Zhai et al. [35] found that DHA and dehydroabietylamine have certain inhibitory activity on *Alternaria brassicae*, *B. cinerea*, *Fusarium solani*, *Fusarium graminearum*. Chen et al. [36] introduced 1,3,4-thiadiazole-thiazolidinone into the DHA skeleton, and synthesized novel DHA derivatives, which showed good antifungal activity against *Gibberella zeae*, with a maximum inhibition rate of 91.3%. Therefore, DHA and its derivatives have the potential to be developed into plant-derived pesticides as well as potential lead compounds for new pesticides due to their excellent and broad-spectrum antimicrobial properties.

The cell membrane, which plays a barrier protection function for pathogenic fungi, is the target of the action of a variety of fungicides [37]. When the cell membrane structure of mycelium is damaged by the external environment, the cell membrane is unable to perform the barrier function, the disruption of cellular homeostasis results in the leakage of intracellular components (including cytoplasm, soluble proteins, sugars, and nucleic acids) into the surrounding medium, which leads to an increase in the extracellular conductivity; thus, changes in conductivity are related to leakage of intracellular components and electrolyte efflux from the mycelium. Yuan et al. [38] found that the treatment of *Colletotrichum gloeosporioides* with rosemary essential oil increased the extracellular conductivity and the leakage of nucleic acids, soluble proteins and soluble reducing sugars of *C. gloeosporioides*, indicating that the plasma membrane and cell wall of the pathogenic fungi were disrupted, thus releasing the cellular contents. Xue et al. [39] found that total alkaloids and berberine of *Coptidis rhizoma* led to metabolic disorders in the fungal by increasing the permeability of the cell membrane, and the greater the membrane permeability, the better the fungal inhibition effect. In the present study, the conductivity and soluble protein content were significantly increased in *A. alternata* after DHA treatment, suggesting that the integrity of *A. alternata* cell membrane was disrupted, and the contents were extravasated, which ultimately led to a reduction in the physiological functions of *A. alternata*.

Increased cell membrane permeability caused by lipid peroxidation is one of the main mechanisms of antifungal agents [40]. MDA is one of the main products of lipid peroxidation, so it can be used to assess whether lipid peroxidation is increased in cell membranes [41]. Lin et al. [42] found that extracts of *Lysurus mokusin* led to oxidative stress in *P. neglecta*, with a large increase in mycelial MDA content, exacerbating cell membrane peroxidation. ROS are substances produced by oxygen metabolism in the body, which are mainly composed of oxygen-containing substances such as H_2_O_2_, OH^−^, O^2−^, etc. At low concentrations, ROS perform signaling functions and are involved in the regulation of organismal growth and development, as well as in responding to external stresses. When a large amount of ROS accumulates, cellular components of organisms such as lipids, nucleic acids, and proteins may be subjected to destructive oxidation, ultimately leading to cell death [43,44,45]. Therefore, in the present study, the MDA and H_2_O_2_ contents in *A. alternata* were significantly increased after DHA treatment, which further reflected the degree of cell membrane damage. DHA may have further increased the degree of lipid peroxidation of fungal cell membranes, and caused some oxidative damage, which led to the accumulation of ROS.

Under normal conditions, the production and removal of ROS in organisms are in a state of dynamic equilibrium, and when subjected to external stress, this equilibrium is disrupted in the cells, and the production of large amounts of ROS leads to oxidative stress, loss of cellular function, and ultimately apoptosis or necrosis [46]. All aerobic organisms have an antioxidant system to control ROS accumulation and maintain redox homeostasis [47]. SOD, CAT, and POD, as key enzymes of the antioxidant system, are critical for promoting ROS scavenging [48]. In this study, SOD, CAT, and POD activities were inhibited in *A. alternata* after treatment with DHA, indicating that the antioxidant capacity of *A. alternata* was decreased, and a large amount of ROS was accumulated in the organism after *A. alternata* was subjected to oxidative damage, and DHA inhibited *A. alternata* by inhibiting the activity of the protective enzymes and decreasing the ability of the pathogenic fungi to scavenge ROS. Similarly to the present study, Bao et al. [49] found that SOD, POD, CAT, SDH, and NAD-MDH activities decreased to varying degrees in *Botrytis cinerea* after piperine treatment, and mycelial viability was decreased and thus inhibited. Pan et al. [50] suggested that matrine exerts antifungal effects by affecting membrane lipid peroxidation and ROS accumulation and attributed the decrease in SOD, CAT, and POD in *Botryosphaeria dothidea* to cellular damage. Therefore, in this study, DHA was able to inhibit the activity of protective enzymes in *A. alternata*, leading to a reduction in the viability of the pathogenic fungus, and this result coincided with the changes in the membrane permeability of the *A. alternata* cells as described above. The overall trend of SOD showed an upward trend, which is likely to be the response of *A. alternata* to the oxidative stress caused by DHA.

The plant cell wall is the first barrier of the plant against pathogenic fungi and consists mainly of cellulose and pectin [51]. Pathogenic fungi successfully infect plants by disrupting the host plant cell wall through the secretion of cell-wall-degrading enzymes [52]. Common enzymes such as cellulases and pectinases are cell-wall-degrading enzymes and their activities is positively correlated with the infestation ability of pathogenic fungi [53,54]. Yang et al. [55] found that the higher the enzyme concentration, the more severe the damage of rice leaves after using cell-wall-degrading enzymes to treat rice leaves. Zhou et al. [56] showed that the crude extract of *Brevibacillus brevis* inhibited both extracellular pectinase and cellulase of *Alternaria solani*, which has potential in green control of tomato early blight. Li et al. [57] investigated the inhibitory effect of phytic acid on three cell-wall-degrading enzymes (EG, PG, PL) of *F. oxysporum*, and it had a good control of seedling blight of *Pinus sylvestris* var. *mongolica*. In this study, the enzyme activities of EG, PG, and PL in *A. alternata* were significantly reduced by DHA, indicating that DHA reduced the ability of *A. alternata* to degrade the cell wall of the host plant, which in turn affected the infestation of the host plant. Therefore, the changes in the incidence area of poplar leaf spot after infestation using *P. alba* leaves further verified that DHA weakened the pathogenicity of *A. alternata*.

The regulation of the expression of genes encoding antioxidant enzymes and cell-wall-degrading enzymes in *A. alternata* by DHA was also investigated. The results showed that the expression of antioxidant enzyme genes *AaSOD*, *AaPOD*, and *AaCAT* as well as cell-wall-degrading enzyme genes *AaPL* and *AaPG* in *A. alternata* were significantly down-regulated in *A. alternata*. Zhang et al. [58] found that *Fusarium pseudograminearum* down-regulated *SOD* and *POD* genes and up-regulated *CAT* gene under the action of the biocontrol agent *Bacillus velezensis* YB-185 by transcriptome analysis, suggesting that *F. pseudograminearum* may maintain normal cellular functions by enhancing antioxidant responses. However, the reduced scavenging activity of free radicals (ABTS+) was determined, indicating that the overall antioxidant capacity was decreased and insufficient to prevent cellular damage. In this study, the down-regulation of three antioxidant enzyme genes also proved that DHA induced oxidative stress in *A. alternata*. In the enzyme activity assay, the activity of SOD showed a gradual increase, but the overall was lower than that of the control group, which also confirmed that *A. alternata* made an antioxidant response to DHA, but the response was insufficient, which still led to the accumulation of free radicals and even cell death. Zhang et al. [59] analyzed the enzyme activity and gene expression of four cell-wall-degrading enzymes of *A. alternata* during the infestation of melons and found that these index quantities were higher in infestation of 86-1 melons. Combined with the melon disease, it indicated that *A. alternata* was more aggressive in infesting 86-1 melon, which shows that the activity and gene expression of cell-wall-degrading enzymes can indicate the infestation ability of the pathogen to a certain extent. Yang et al. [60] found that the expression of three cell-wall-degrading enzyme-related genes (*PnEG*, *PnBG* and *PnPG*) were inhibited in *P. neglecta* after 60 min of action using different concentrations of sodium pheophorbide a treatment. Paccanaro et al. [61] found that the pathogenicity of *F. graminearum* on wheat was significantly reduced when *xyrl*, a major regulatory gene encoding PG, was knocked out. Therefore, in this study, it is likely that DHA affects the pathogenicity of *A. alternata* by inhibiting the expression of the genes for these cell-wall-degrading enzymes, while the changes in the expression of this range of genes confirmed the enzyme activity results.

## 5. Conclusions

The present study revealed the fungicidal activity and mechanism of DHA against *A. alternata* and identified the potential of DHA to be developed as a plant-derived antifungal agent. DHA could destroy the morphology of *A. alternata* mycelium, increase the permeability of the cell membrane, lead to a large leakage of intracellular substances, and at the same time cause oxidative damage to the mycelial cells, inhibit the activities of the defense-related enzymes and pathogenicity-related enzymes in the pathogenic fungi, reduce the viability and pathogenicity of *A. alternata*, and thus exert antifungal activity. Therefore, it is of practical significance to develop DHA into a new low-toxicity and high-efficiency plant-derived pesticide for the control of poplar leaf spot caused by *A. alternata*.

## Figures and Tables

**Table 1:** Primer sequence.

Primer Name	Gene ID	Encoded Protein	Forward Primer (5′−3′)	Reverse Primer (5′−3′)
*AaSOD*	29119212	Cu/Zn superoxide dismutase	TCCTCGCAACCGCAAAGTCT	CTTGAGCCCTCCCGTCTCAC
*AaPOD*	29116333	Peroxidase	AGGAAGGACCAGACAAGCAC	ATCTTCCTGCTGCTGGTCTG
*AaCAT*	29114347	Catalase	ATGGTGCGAGTTCAGATCCT	CGTCAGTCGACGCAGCATTG
*AaPL*	29114697	Pectin lyase	GGATGTTTGCATGAACTACT	CTATCTGCAGACGGGAAGGG
*AaPG*	29119730	Polygalacturonase	ATGGTTGCCTTTGCACTCAC	TGCAAGAAGCCTTGCCCTTG
*AaBenA*	29108751	β-tubulin	GTTGAGAACTCAGACGAGACCTTCTGCATTG	CTCCTTCAACCGGCAGTTGTACCAAG

**Table 2:** Toxicity equations of dehydroabietic acid against five pathogenic fungi.

Phytopathogenic Fungi	Toxicity Equation	X^2^	R^2^	EC_30_ (mg/L)	95% Confidence Interval (mg/L)	EC_50_ (mg/L)	95% Confidence Interval (mg/L)
*A. alternata*	y = 0.7x − 1.23	1.903	0.991	9.966	6.672–13.691	56.015	45.542–67.604
*P. neglecta*	y = 0.94x − 2.41	4.227	0.988	101.918	87.669–117.725	492.687	306.352–441.426
*F. oxysporum*	y = 0.69x − 1.92	13.724	0.935	106.78	87.701–129.194	595.547	456.929–827.609
*B. cinerea*	y = 0.64x − 1.18	4.062	0.979	10.727	6.969–15.009	70.664	57.146–86.211
*V. mali*	y = 0.76x − 1.42	20.814	0.924	14.763	10.686–19.21	72.319	60.488–85.681

## Data Availability

The original contributions presented in this study are included in the article. Further inquiries can be directed to the corresponding author.

## Abbreviations

The following abbreviations are used in this manuscript:

DHA	Dehydroabietic acid
RT-qPCR	Real-time quantitative polymerase chain reaction
SOD	Superoxide dismutase
CAT	Catalase
POD	Peroxidase
EG	Endoglucanase
PG	Polygalacturonase
PL	Pectin lyase
PDA	Potato dextrose agar
MDA	Malondialdehyde
H_2_O_2_	Hydrogen peroxide
DMSO	Dimethyl sulfoxide
SEM	Scanning electron microscopy
RNA	Ribonucleic acid
